# CCN2 Increases TGF-β Receptor Type II Expression in Vascular Smooth Muscle Cells: Essential Role of CCN2 in the TGF-β Pathway Regulation

**DOI:** 10.3390/ijms23010375

**Published:** 2021-12-29

**Authors:** Antonio Tejera-Muñoz, Laura Marquez-Exposito, Lucía Tejedor-Santamaría, Sandra Rayego-Mateos, Macarena Orejudo, Beatriz Suarez-Álvarez, Carlos López-Larrea, Marta Ruíz-Ortega, Raúl R. Rodrigues-Díez

**Affiliations:** 1 Molecular and Cellular Biology in Renal and Vascular Pathology, IIS-Fundación Jiménez Díaz-Universidad Autónoma Madrid, 28040 Madrid, Spain; antoniotemu@gmail.com (A.T.-M.); laura.marqueze@quironsalud.es (L.M.-E.); luciatejedors@hotmail.com (L.T.-S.); srayego@fjd.es (S.R.-M.); macaorejudo@gmail.com (M.O.); 2 Red de Investigación Renal (REDINREN), Instituto de Salud Carlos III, 28029 Madrid, Spain; bea230@hotmail.com (B.S.-Á.); inmuno@hca.es (C.L.-L.); 3 Translational Immunology Laboratory, Health Research Institute of Asturias (ISPA), 33011 Oviedo, Spain; 4 Department of Immunology, Hospital Universitario Central De Asturias, 33011 Oviedo, Spain

**Keywords:** CCN2, TGF-β, SMAD, TGF-β receptors, EGFR, CTGF

## Abstract

The cellular communication network factor 2 (CCN2/CTGF) has been traditionally described as a mediator of the fibrotic responses induced by other factors including the transforming growth factor β (TGF-β). However, several studies have defined a direct role of CCN2 acting as a growth factor inducing oxidative and proinflammatory responses. The presence of CCN2 and TGF-β together in the cellular context has been described as a requisite to induce a persistent fibrotic response, but the precise mechanisms implicated in this relation are not described yet. Considering the main role of TGF-β receptors (TβR) in the TGF-β pathway activation, our aim was to investigate the effects of CCN2 in the regulation of TβRI and TβRII levels in vascular smooth muscle cells (VSMCs). While no differences were observed in TβRI levels, an increase in TβRII expression at both gene and protein level were found 48 h after stimulation with the C-terminal fragment of CCN2 (CCN2(IV)). Cell pretreatment with a TβRI inhibitor did not modify TβRII increment induced by CCN2(VI), demonstrating a TGF-β-independent response. Secondly, CCN2(IV) rapidly activated the SMAD pathway in VSMCs, this being crucial in the upregulation of TβRII since the preincubation with an SMAD3 inhibitor prevented it. Similarly, pretreatment with the epidermal growth factor receptor (EGFR) inhibitor erlotinib abolished TβRII upregulation, indicating the participation of this receptor in the observed responses. Our findings suggest a direct role of CCN2 maintaining the TGF-β pathway activation by increasing TβRII expression in an EGFR-SMAD dependent manner activation.

## 1. Introduction

The transforming growth factor beta (TGF-β) belongs to the TGF-β growth factor superfamily implicated in cell division, differentiation, migration, adhesion, organization and death [1,2,3]. The relevant role of TGF-β in cellular homeostasis has been widely demonstrated and the deregulation of their related pathways has been associated with several human pathologies including cancer, autoimmune disorders and cardiovascular diseases [4,5]. The classical TGF-β pathway activation starts with TGF-β binding to its serin-treonin kinase receptor type II (TβRII) which leads to receptor type I (TβRI) activation. The active TβRI, enlists and phosphorylates the receptor-regulated SMADs proteins (R-SMADs: SMAD 2 and SMAD 3) inducing the formation of a new heterocomplex with the common-mediator SMAD (SMAD 4). Finally, this R-SMAD/SMAD4 complex is translocated into the nucleus regulating the expression of their related genes through the interaction with other transcription factors [4,6,7]. Aside from this classical activation, other different pathways activated by TGF-β have also been described, including ERK, p38 mitogen activated protein kinase (MAPK), JNK and PI3K/AKT cascades [8,9].

Regarding cardiovascular system, TGF-β pathway plays an essential role in the vascular wall formation during the embryonic development [5,10]. In mature vessels, TGF-β has been directly related with the development of vascular fibrosis [11,12]. However, it has been also described as a vascular anti-inflammatory mediator [13,14]. Interestingly, although the anti-inflammatory effects were largely demonstrated in vascular smooth muscle cells (VSMCs), a pro-inflammatory role has been recently attributed to the endothelial-derived TGF-β in atherosclerosis lesions [15]. These results demonstrate the complexity of the TGF-β pathway regulation, and encourage new studies to further unravel the processes involved.

Among the large number of genes regulated by TGF-β, one of the most increased factors already described is the Cellular Communication Network Factor 2 (CCN2), previously known as connective tissue growth factor (CTGF) [16]. CCN2 is a matricellular protein encompassed in the CCN protein family, composed also by CCN1/Cyr61 (cysteine rich protein), CCN3/Nov (nephroblastoma overexpressed protein) and other three secreted proteins (CCN4-6) [17]. CCN2 is implicated in several biological processes such as cell proliferation, survival, angiogenesis and migration [18,19,20]. As other CCN members, CCN2 are composed by four distinct modules: 1: insulin-like growth factor binding protein; 2: von Willebrand factor type C repeat; 3: thrombospondin type 1 repeat; and 4: C-terminal cystine-knot [21]. CCN2 is secreted as a preprotein that requires proteolytic processing to release the C-terminal domains and to be fully biologically active [22]. Increased levels of CCN2 have been described in several pathologies, including cardiovascular diseases like heart failure, pulmonary hypertension, vascular remodeling and atherosclerosis [20,23,24,25]. Traditionally, CCN2 has been described as a downstream mediator of the profibrotic responses induced by other factors such as the above mentioned TGF-β, endothelin or angiotensin II (Ang II) in VSMCs [11,26,27]. Nevertheless, during last years, our group, and many others, described the ability of the CCN2-C-terminal module (CCN2-IV) to directly induce and activate pro-oxidative and pro-inflammatory responses [28,29,30,31]. Increased circulating CCN2 levels have been suggested as a risk biomarker for cardiac dysfunction in patients with myocardial fibrosis and chronic heart failure [32]. However, at experimental level, there is different evidence depending on the pathology. In this sense, while CCN2 blockade have shown beneficial effects in experimental aortic restenosis, pulmonary vascular remodeling [24,33] or in several models of liver, lung and kidney fibrosis in mice [34,35,36], CCN2 overexpression demonstrated cardio-protective effects in Ang-II infused mice after experimental myocardial injury induced by ischemia reperfusion [37,38,39,40,41].

The essential participation of CCN2 in the TGF-β profibrotic responses has been described in some studies. Thereby, in dermal and lung fibroblast CCN2 is necessary to the maintenance of a persistent fibrotic tissue formation induced by TGF-β [42,43]. Accordingly, although CCN2 by itself is not considered a direct fibrotic factor, their presence is crucial to generate a fibrotic environment in experimental lung fibrosis in mice [36,44,45,46]. These results suggest the existence of other mechanisms directly induced by CCN2 that may contribute to the maintenance of the fibrotic response elicited by TGF-β.

Despite this close relation between TGF-β and CCN2, the potential downstream regulation of the TGF-β pathway by CCN2 remains unclear. Considering the main role of the TFG-β receptors in the TGF-β pathway activation and its maintenance [47,48,49], our aim was to evaluate whether CCN2 modulate TGF-β receptors levels in VSMCs and to describe the potential mechanisms implicated.

## 2. Results

### 2.1. CCN2(IV) Increases TβRII Levels in Cultured VSMCs after 48 h

**Figure 1:**
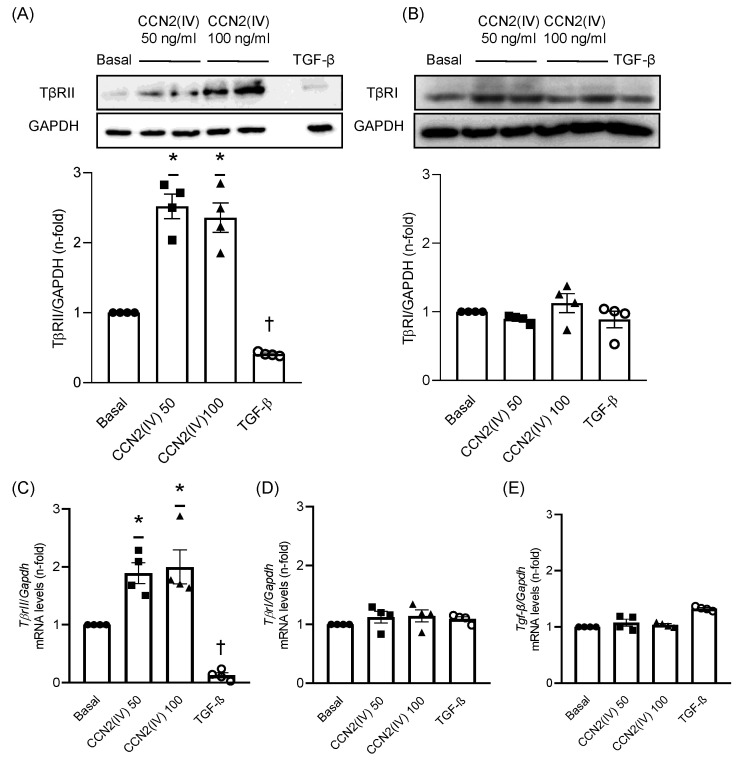
TβRII protein and mRNA levels are increased by CCN2(IV) stimulus in VSMCs in vitro. CCN2(IV)-incubated vascular smooth muscle cells (VSMCs) showed increased protein levels of TβRII at doses of 50 ng/mL and 100 ng/mL after 48 h of treatment, whereas TGFβ (1 ng/mL) treatment decreased TβRII protein levels compared to non-treated VSMCs (**A**). Protein levels of TβRI were not modified neither by CCN2(IV) nor TGFβ (**B**). After 48 h of treatment, CCN2(IV) increased *TβrII* (**C**), but not *TβrI* (**D**) and *Tgf-β* (**E**) mRNA levels, whilst TGF-β stimulation decreased *TβrII* and did not modify *TβrI* and *Tgf-β* mRNA levels. Data are presented as mean ±SEM of 4 independent experiments. * *p* < 0.05 increased vs. Basal; † *p* < 0.05 decreased vs. Basal.

In order to evaluate whether CCN2 could modulate TGF-β receptors levels, culture VSMCs were treated with the recombinant CCN2(IV) at different doses (50 or 100 ng/mL). CCN2(IV) significantly increased TβRII protein levels in VSMCs at both doses compared with basal condition cells after 48 h (Figure 1A). Conversely, the treatment with TGF-β (10 ng/mL) induced a significant decrease in the TβRII levels (Figure 1A). On the other hand, neither CCN2(IV) nor TGF-β modified TβRI protein levels (Figure 1B). Similarly, while CCN2(IV) also increased TβRII mRNA levels after 48 h at 50 and 100 ng/mL doses, TGF-β dramatically decrease them (Figure 1C). Finally, CCN2(IV) treatment did not modify TβRI and TGF-β mRNA levels at any studied dose after 48 h (Figure 1D,E).

### 2.2. CCN2(IV) Activates the SMAD Pathway in Cultured and Aortic VSMCs at Short Times

**Figure 2:**
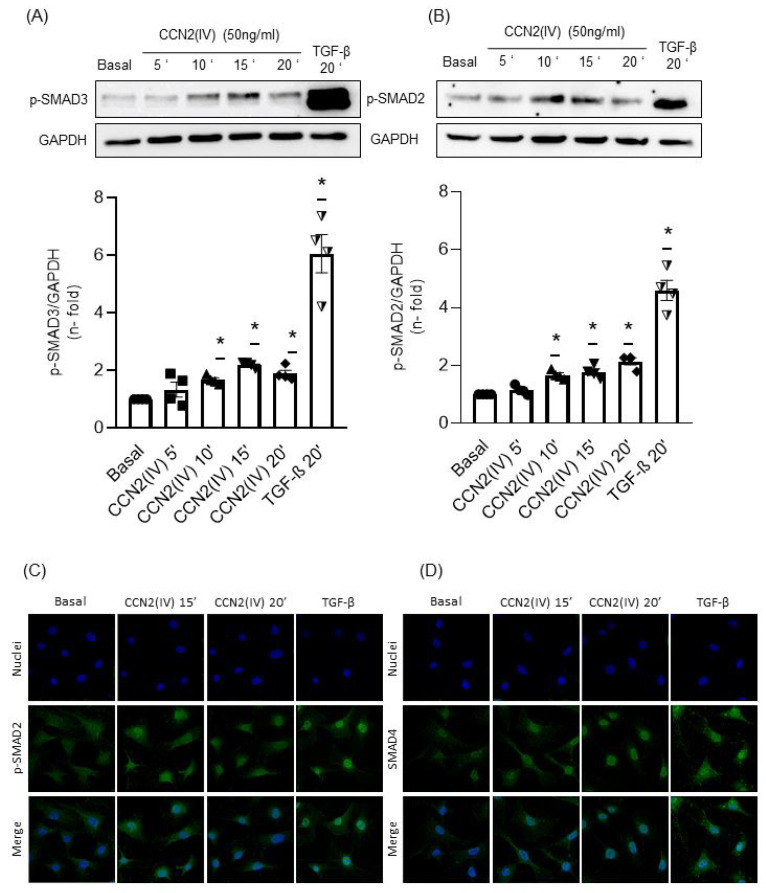
CCN2(IV) promotes SMAD pathway activation by phosphorylation of SMAD2 and SMAD3 in vitro. CCN2(IV) (50 ng/mL) induced SMAD3 (**A**) and SMAD2 (**B**) phosphorylation in VSMCs at early time-points (5 to 20 min) represented as p-SMAD3 and p-SMAD2 respectively. These results agreed with an increase in the nuclear translocation of p-SMAD2 (**C**) and SMAD4 (**D**) after 10 and 20 min of treatment with CCN2(IV) in VSMCs. Data are presented as mean ± SEM of 4 independent experiments. * *p* < 0.05 increased vs. Basal.

**Figure 3:**
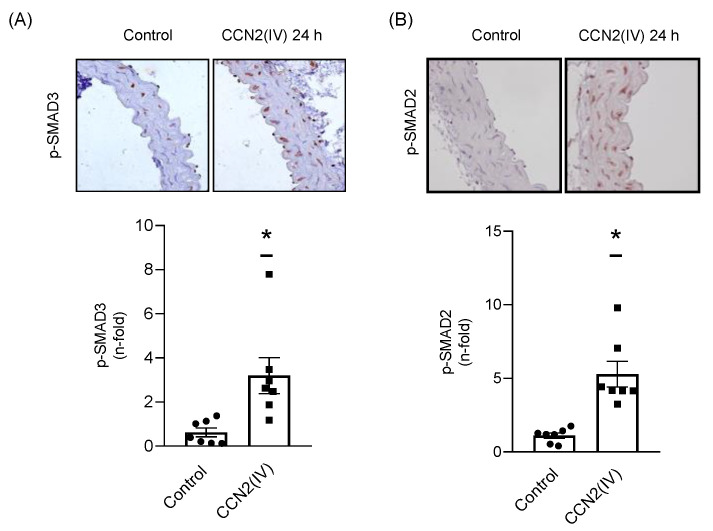
p-SMAD3 and p-SMAD2 are higher expressed in aortic VSMCs from mice injected with CCN2(IV). Intraperitoneal administration of CCN2(IV) (2.5 ng/g) increased aortic levels of p-SMAD3 (**A**) and p-SMAD2 (**B**) compared to control group after 24 h of treatment. Microphotographs show aorta immunohistochemistry (40× magnification) of both groups. Data are presented as mean ± SEM of 7 mice per group. * *p* < 0.05 increased vs. Basal.

To test whether CCN2 directly activate the SMAD pathway, culture VSMCs were incubated with CCN2(IV) at different times. After 10, 15 and 20 min, CCN2(IV) induced a significant SMAD pathway activation observed by increased levels of phosphorylated SMAD3 (Figure 2A, p-SMAD3) and SMAD2 (Figure 2B, p-SMAD2). The SMAD pathway activation was confirmed by translocation of p-SMAD2 (Figure 2C) and SMAD 4 (Figure 2D) into the VSMCs nuclei after 10 and 20 min. As observed in vitro, the in vivo intraperitoneal CCN2(IV) administration in mice increased p-SMAD3 (Figure 3A) and p-SMAD2 (Figure 3B) levels in the aortic VSMCs after 24 h. In this set of experiments, TGF-β (1 ng/mL) was used as positive control.

### 2.3. CCN2(IV) Increases TβRII Expression in VSMCs by TGF- β –Independent SMAD Activation

**Figure 4:**
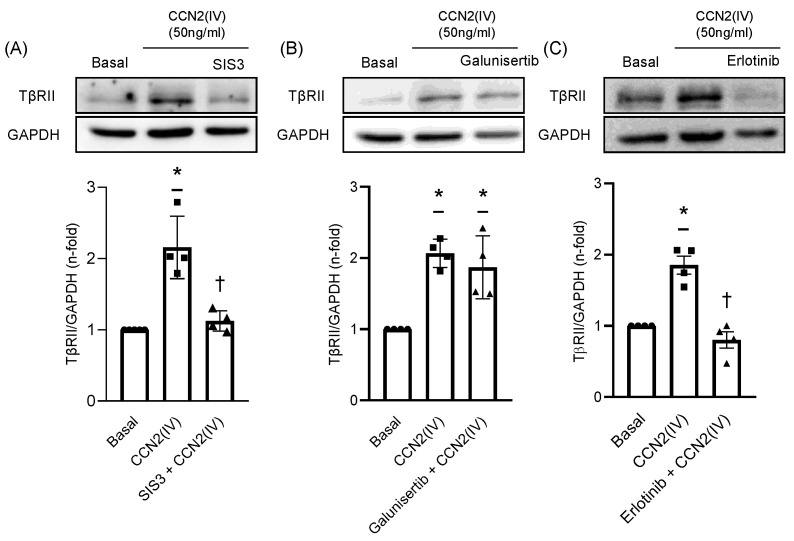
CCN2(IV) triggers TβRII expression via EGFR/SMAD activation and independently of TGFβ pathway in VSMCs in vitro. The increased expression of TβRII induced by CCN2(IV) after 48 h was significantly reduced in VSMCs preincubated 1 h with SIS3, a pharmacological inhibitor of the SMAD3 activation (**A**). However, 1 h of preincubation with galunisertib, a pharmacological inhibitor of TβRI, did not prevent TβRII overexpression induced by CCN2(IV) in VSMCs (**B**). On the other hand, preincubation of VSMCs with erlotinib, a pharmacological inhibitor of EGFR, 1 h before CCN2(IV) addition, significantly prevented TβRII overexpression after 48 h (**C**). Data are presented as mean ± SEM of 4 independent experiments. * *p* < 0.05 increased vs. Basal; † *p* < 0.05 decreased vs. Basal.

The potential role of SMAD pathway activation induced by CCN2(IV) in the regulation of TβRII expression was evaluated by using the SMAD3 inhibitor SIS3. The protein levels evaluation demonstrated that preincubation of VSMCs with SIS3 for 1 h inhibited TβRII upregulation induced by CCN2(IV) after 48 h of treatment (Figure 4A), demonstrating a direct role of SMAD activation in this process. On the other hand, preincubation of VSMCs for 1 h with galunisertib, a potent TβRI inhibitor, did not modulate TβRII levels in CCN2(IV) treated cells (Figure 4B), indicating a TGF-β independent response.

### 2.4. TβRII Expression Induced by CCN2(IV) in VSMCs Is Mediated by the EGF Receptor

Considering our previous published results describing the ability of CCN2 to directly bind to and activate the epidermal growth factor receptor (EGFR) [28,50], the next aim was to determine the participation of this receptor in the observed results. The preincubation of VSMCs with the EGFR inhibitor erlotinib 1-h prior CCN2(IV) administration, prevented TβRII upregulation after 48 h (Figure 4C).

## 3. Discussion

The present study points out a direct role of CCN2 increasing TβRII levels and, therefore, suggests its participation exerting positive feedback in the TGF-β pathway activation. On the contrary, TGF-β induces a reduction in TβRII levels, indicating the complexity of this pathway regulation. Our results provide a potential explanation of the previously described relevance of CCN2 maintaining the TGF-β profibrotic response and open new ways to future studies in this field.

The regulation of the TGF-β pathway comprises a wide range of components and factors, which lead into the activation of a large list of genes [51]. Among the latter, CCN2 plays an essential role in the profibrotic response induced by TGF-β [52,53]. TGF-β pathway components levels have been described to be essential in the regulation of the TGF-β activation [48]. Upon their activation, the heteromeric TβRI/TβRII complexes are rapidly internalized into the cytoplasm by, at least, two different processes: the classical clathrin-dependent pathway, which helps SMAD activation, and a lipid raft-caveolin dependent process which mediates the receptor degradation [54]. Consequently, the specificity of signaling pathway activation and the biological effects of TGF-β are modulated by TGF-β receptor levels [47]. At vascular level, the relevance of TGF-β receptors has been described in several pathologies. Thus, the cell-phenotype conversion from an antiproliferative to a profibrotic response after TGF-β stimulation observed in VSMCs derived from human atherosclerotic and restenotic lesions, was attributed to the decreased ratio of TβRII/TβRI [55,56,57]. Interestingly, one of the most used drugs for atherosclerosis treatment, statins, have demonstrated to increase TβRII expression, as well as CCN2 production in cultured VSMCs [58]. Consequently, atorvastatin treatment increased TβRII expression in the atheroma plaque in an experimental model of atherosclerosis in Apolipoprotein E Knockout mice, which was associated to beneficial effects, including amelioration of disease progression and stabilization of the atheroma plaque by increased CCN2 expression and collagen content [58]. These data suggest an interrelation between CCN2 and TβRII regulation both in vitro and in vivo in VSMCs. Here we demonstrate that CCN2 increased TβRII expression in cultured VSMCs at both protein and gene level after 48 h, while TβRI levels remained unaltered. Contrarily, TGF-β decreased TβRII levels after 48 h of stimulation, which could correspond to the above-mentioned TGF-β receptor degradation. These findings suggest a direct role of CCN2 maintaining positive feedback in the TGF-β response.

**Figure 5:**
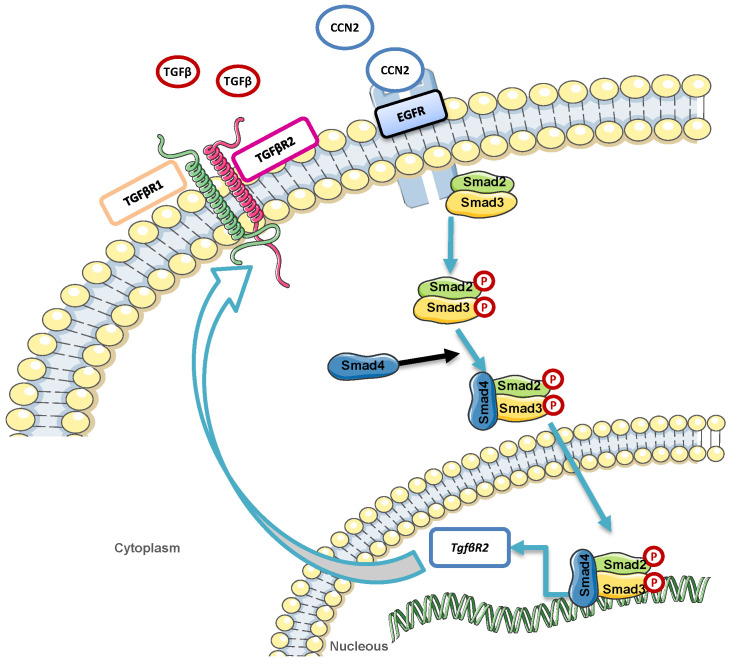
Graphical scheme of proposed mechanism by which CCN2 may be triggering the expression of TβRII, compared to canonical TGF-β pathway activation.

CCN2 exerts a dual role in the vasculature not only acting as a growth factor, but also maintaining vascular homeostasis [22,59,60]. This feature could explain the different results obtained modulating CCN2 levels in experimental cardiovascular pathologies showing the benefits of both blocking [24,61,62] or overexpressing CCN2 [37,38,40,41,63], depending on the disease. Regarding gene expression regulation, CCN2 knockout mice die shortly after birth by respiratory failure due to its essential role in coordinating chondrogenesis and angiogenesis during skeletal development [64]. In adult mice, tamoxifen-dependent CCN2 deletion ameliorated renal fibrosis [65], but it did not improve cardiac fibrosis and hypertrophy [66]. Recently, our group has demonstrated the relevance of CCN2 on maintaining vascular wall homeostasis in a model of Ang II-induced vascular damage. In this sense, acquired CCN2 deletion in adult mice predispose to rapid aortic aneurysms development and rupture after Ang II administration [67]. These results are similar to those observed in experimental mice models combining TGF-β neutralization with Ang II infusion, which enhanced AngII-induced aortic rupture and aneurysm in both thoracic and abdominal regions [68,69]. Although further studies are necessary to further evaluate this hypothesis, the present results open new potential mechanisms in which CCN2, by increasing TβRII expression in VSMCs, could exert positive feedback in the TGF-β pathway activation, contributing to the TGF-β-beneficial effects described in some vasculopathy situations [70]. Regarding CCN2-growth factor actions, our previous studies described the ability of CCN2 to induce pro-oxidative and pro-inflammatory responses in cultured VSMCs and mice aorta [28]. In the present study we demonstrated that CCN2 also activates SMAD pathway in cultured VSMCs at early time-points leading to TβRII production after 48 h. We have previously described that CCN2 directly binds to and activates EGFR [28,50] to induce pro-oxidative and pro-inflammatory responses in VSMCs. In this new study we further strengthen the EGFR participation in CCN2 responses, demonstrating that this receptor is essential to induce TβRII overexpression mediated by CCN2(IV) in VSMCs. Altogether our data suggest a potential mechanism implicated in TβRII overexpression induced by CCN2(IV) that include EGFR and SMAD pathway activation (Figure 5).

## 4. Materials and Methods

### 4.1. Experimental Mice Model

Experimental animal studies were performed in adult male C57BL/6 mice (9–12 weeks old, 20 g; Harlan Interfauna lbérica, S.A., Barcelona, Spain) and maintained in the animal facilities of the “Instituto de Investigación Sanitaria Fundación Jiménez Díaz” (IIS-FJD) fed with standard diet and water ad libitum, under special pathogen-free conditions and normal light-dark cycles. All the procedures with animals were performed according to the European Community (RD53/2013) and IIS-FJD Animal Research Ethical Committee guidelines (PROEX 065/18). CCN2(IV) administration was performed as previously described [28]. Briefly, mice were intraperitoneally injected with CCN2(IV) (2.5 ng/g of body weight, dissolved in saline) and were euthanatized after 24 h under anesthesia (Isofluorane; Abbott Laboratories, Madrid, Spain). Aortas were collected, dissected free of fat and connective tissue, fixed in paraformaldehyde and embedded in paraffin. A control saline-injected group was also studied (*n* = 7 mice per group). The purity of CCN2(IV) (endotoxin levels < 0.01) was evaluated by MALDI-TOF (data not shown).

### 4.2. Histological Analysis

Aortic sections embedded in paraffin were placed in coated slides (4 µm thick), deparaffinized, rehydrated (alcohol gradient from xylene, alcohol 100-95-70%) and washed in distilled water. For immunostaining, antigens were restored by using PTLink system (DAKO Diagnosticos, Barcelona, Spain), blocking endogenous peroxidase afterwards. Commercial casein solution (DAKO Diagnosticos) was used to release non-specific protein bindings (1 h at room temperature) and tissue sections were incubated overnight at 4 °C with primary p-SMAD2 ([1/200]; #3108, Cell signaling, Danvers, MA, USA) or p-SMAD3 ([1/200]; ab52903, Abcam, Cambridge, UK) antibody diluted in antibody solution (DAKO Diagnosticos). Sections were incubated with the specific HRP secondary antibodies (GENA934, Sigma Chemical, St. Louis, MO, USA) for 1 h followed by Avidin-Biotin Complex incubation (Vector laboratories, Burlingame, CA, USA) during 30 min. To develop signal, samples were incubated with substrate solution and 3,3-diaminobenzidine as a chromogen (Abcam, Cambridge, UK) and counterstained with Carazzi’s haematoxylin (Thermo Fisher Scientific, Rockford, IL, USA). For each antibody, specificity was checked by omission of primary antibodies (data not shown). Quantification was made by using the Image-Pro Plus software (Bio-Rad, Hercules, CA, USA) determining the positive relative staining area per total area in 5 to 10 randomly chosen fields (×40 magnification) taken with LeicaDMD108 microscope (Leica Microsystem, Wetzlar, Germany).

### 4.3. Cell Cultures

Vascular smooth muscle cells (VSMCs) came from mice aorta cell line MOVAS (ATCC CRL-2797; Barcelona, Spain). VSMCs were cultured in Dulbecco’s modified Eagle’s medium (DMEM) supplemented with 10% fetal bovine serum (FBS), 2% L-glutamine 200 mM, 100 U/mL penicillin, 100 U/mL streptomycin and 0.2 mg/mL G-418 (all reagents were obtained from Sigma Chemical, MO, USA). Every experiment was performed at 80% of confluence, as well as in growth-arrested cells conditioned by serum starvation during the 24 h prior to stimuli. Cells were treated with recombinant C-terminal CCN2 (CCN2(IV)) (Peprotech, London, UK) as stimulus at concentrations of 50 and 100 ng/mL evaluated at different time-points. According to previous studies of our group [58,71], SMAD pathway activation was evaluated at early time-points (5 to 20 min) whereas TGF-β receptors levels were assessed after 48 h of CCN2(IV) treatment. The following pharmacological inhibitors were used to study different pathways: SIS3 (Selleck Chemicals, Berlin, Germany), as a SMAD3 phosphorylation inhibitor [72], galunisertib (Selleck Chemicals) a TβRI antagonist [73], and erlotinib, an EGFR inhibitor (Vichem Chemie Research, Budapest, Hungary).

### 4.4. Immunofluorescence

Immunofluorescence studies were assessed in VSMCs seeded in 24-well Multidish over glass coverslips (Cultek, Madrid, Spain). Once experiments were done, cells were fixed in 4% PFA, treated with 0.1% Triton-X100 and blocked with 4% BSA in TBS. Afterwards, cells were incubated with p-SMAD2 ([1/200]; #3108, Cell signaling, MA, USA) and p-SMAD4 antibodies ([1/200]; sc7966; Santa Cruz Biotechnology, Heidelberg, Germany) overnight, followed by 1 h of incubation with AlexaFluor^®^488 conjugated secondary antibody (1/300; Invitrogen, Life Technologies, Philadelphia, PA, USA). DAPI was used as nuclear counterstaining (Sigma Chemical). Negative control was also performed in absence of primary antibody (data not shown) in order to verify specificity of the immunostaining. Finally, samples were mounted in ProlongGoldTM (Invitrogen, Life Technologies) and visualized in a Leica DM-IRB confocal microscope.

### 4.5. qPCR Analysis

Total mRNA was obtained by using TRIzol method as previously described (Invitrogen) and retro-transcribed into cDNA by using the Reverse Transcription kit (Applied biosystems, Life Technologies, Inchinnan, UK). Multiplex real time-PCR was performed using fluorogenic primers designed by the Assay-on-Demand mouse gene expression products (Applied Biosystems): *Tgfb* (Mm01178820_m1), *Tgfbr1* (Mm00436964_m1) and *Tgfbr2* (Mm03024091_m1) (FAM). Glyceraldehyde 3-phosphate dehydrogenase (*Gapdh*) (Mm99999915_g1, VIC) was used as endogen control to normalized data. The mRNA copy number were calculated for each sample by the instrument software (ABIPrism 7500 Fast sequence detection PCR system software (Applied Biosystems)) using Ct value (“arithmetic fit point analysis for the lightcycler”), and results were expressed in n-fold calculated vs. control.

### 4.6. Western Blot

Total proteins were isolated from the whole frozen aorta using lysis buffer as previously described [28]. Afterwards, proteins were quantified using bicinchoninic acid assay (BCA) method (Thermo Fisher Scientific). A total 50 ug of proteins were loaded and separated on 10% polyacrylamide-SDS gels under reducing conditions. At the end of electrophoresis, proteins were transferred to nitrocellulose membranes (Amersham Bioscience, Buckinghamshire, UK). Membranes were blocked in TBS containing 0.1% Tween20 (TBST) and 5% dry non-fat milk (1 h at room temperature) and incubated with the different primary antibodies overnight at 4 °C. Next day, membranes were washed 10 min three times with TBST and incubated 1 h with the appropriate HRP (horseradish peroxidase)-conjugated secondary antibody (anti-rabbit, GENA934, anti-mouse, GENA931, Sigma Chemical [1/2000]) at room temperature. ECL kit (Amersham Bioscience) was used to develop. Results were analyzed by LAS 4000 (GE Healthcare Systems, Chicago, IL, USA) and the quantification of the bands density was done by using the Quantity One software (Bio-Rad, CA, USA). The following primary antibodies were employed p-SMAD2 (#3108, Cell signaling (1/1000)), p-SMAD3 (ab52903; abcam; (1/1000)), TβRII ((sc17792; Santa Cruz Biotechnologies; 1/300)) and TβRI (sc518086; Santa Cruz Biotechnologies; [1/300]).

### 4.7. Statistical Analysis

Data are expressed as mean ± standard error of the mean (SEM). Normality distribution was tested by using Shapiro-Wilk test. If the samples followed a Gaussian distribution or not, means were compared by t-student or Mann-Whitney statistical test respectively. Every statistical analysis was conducted using GraphPad Prism 8.0 (GraphPad Software, San Diego, CA, USA). Values of *p* < 0.05 were considered statistically significant.

## 5. Conclusions

The present study contributes to extend the complex mechanism implicated in the TGF-β pathway regulation suggesting that CCN2 expression induced by TGF-β positively regulates TβRII synthesis, which could compensate TβRII degradation induced by TGF-β and, therefore, explain the essential role of CCN2 maintaining the TGF-β-mediated profibrotic responses.

## Data Availability

Not applicable.

## References

[1] Javelaud D., Mauviel A. (2004). Mammalian transforming growth factor-betas: Smad signaling and physio-pathological roles. Int. J. Biochem. Cell Biol..

[2] Massagué J., Seoane J., Wotton D. (2005). Smad transcription factors. Genes Dev..

[3] Moustakas A., Pardali K., Gaal A., Heldin C.H. (2002). Mechanisms of TGF-beta signaling in regulation of cell growth and differentiation. Immunol. Lett..

[4] Batlle E., Massagué J. (2019). Transforming Growth Factor-β Signaling in Immunity and Cancer. Immunity.

[5] Goumans M.J., ten Dijke P. (2018). TGF-β Signaling in Control of Cardiovascular Function. Cold Spring Harb. Perspect. Biol..

[6] Tzavlaki K., Moustakas A. (2020). TGF-β Signaling. Biomolecules.

[7] Leask A. (2008). Targeting the TGFβ, endothelin-1 and CCN2 axis to combat fibrosis in scleroderma. Cell. Signal..

[8] Moustakas A., Heldin C.H. (2005). Non-Smad TGF-beta signals. J. Cell Sci..

[9] Zhang Y.E. (2009). Non-Smad pathways in TGF-beta signaling. Cell Res..

[10] Goumans M.J., Liu Z., Ten Dijke P. (2009). TGF-beta signaling in vascular biology and dysfunction. Cell Res..

[11] Ruiz-Ortega M., Rodriguez-Vita J., Sanchez-Lopez E., Carvajal G., Egido J. (2007). TGF-beta signaling in vascular fibrosis. Cardiovasc. Res..

[12] Cichon M.A., Radisky D.C. (2014). Extracellular matrix as a contextual determinant of transforming growth factor-β signaling in epithelial-mesenchymal transition and in cancer. Cell Adh. Migr..

[13] Li M.O., Wan Y.Y., Sanjabi S., Robertson A.K.L., Flavell R.A. (2006). Transforming growth factor-beta regulation of immune responses. Annu. Rev. Immunol..

[14] Toma I., McCaffrey T.A. (2012). Transforming growth factor-β and atherosclerosis: Interwoven atherogenic and atheroprotective aspects. Cell Tissue Res..

[15] Chen P.Y., Qin L., Li G., Wang Z., Dahlman J.E., Malagon-Lopez J., Gujja S., Cilfone N.A., Kauffman K.J., Sun L. (2019). Endothelial TGF-β signalling drives vascular inflammation and atherosclerosis. Nat. Metab..

[16] Perbal B., Tweedie S., Bruford E. (2018). The official unified nomenclature adopted by the HGNC calls for the use of the acronyms, CCN1–6, and discontinuation in the use of CYR61, CTGF, NOV and WISP 1–3 respectively. J. Cell Commun. Signal..

[17] Perbal A., Perbal B. (2016). The CCN family of proteins: A 25th anniversary picture. J. Cell Commun. Signal..

[18] De Winter P., Leoni P., Abraham D. (2008). Connective tissue growth factor: Structure-function relationships of a mosaic, multifunctional protein. Growth Factors.

[19] Perbal B. (2004). CCN proteins: Multifunctional signalling regulators. Lancet.

[20] Ponticos M. (2013). Connective tissue growth factor (CCN2) in blood vessels. Vascul. Pharmacol..

[21] Leask A., Abraham D.J. (2006). All in the CCN family: Essential matricellular signaling modulators emerge from the bunker. J. Cell Sci..

[22] Kaasbøll O.J., Gadicherla A.K., Wang J.H., Monsen V.T., Hagelin E.M.V., Dong M.Q., Attramadal H. (2018). Connective tissue growth factor (CCN2) is a matricellular preproprotein controlled by proteolytic activation. J. Biol. Chem..

[23] Cicha I., Yilmaz A., Klein M., Raithel D., Brigstock D.R., Daniel W.G., Goppelt-Struebe M., Garlichs C.D. (2005). Connective tissue growth factor is overexpressed in complicated atherosclerotic plaques and induces mononuclear cell chemotaxis in vitro. Arterioscler. Thromb. Vasc. Biol..

[24] Kundi R., Hollenbeck S.T., Yamanouchi D., Herman B.C., Edlin R., Ryer E.J., Wang C., Tsai S., Liu B., Kent K.C. (2009). Arterial gene transfer of the TGF-beta signalling protein Smad3 induces adaptive remodelling following angioplasty: A role for CTGF. Cardiovasc. Res..

[25] Oemar B.S., Werner A., Garnier J.M., Do D.D., Godoy N., Nauck M., März W., Rupp J., Pech M., Lüscher T.F. (1997). Human connective tissue growth factor is expressed in advanced atherosclerotic lesions. Circulation.

[26] Rodriguez-Vita J., Ruiz-Ortega M., Rupérez M., Esteban V., Sanchez-López E., Plaza J.J., Egido J. (2005). Endothelin-1, via ETA receptor and independently of transforming growth factor-β, increases the connective tissue growth factor in vascular smooth muscle cells. Circ. Res..

[27] Rupérez M., Lorenzo Ó., Blanco-Colio L.M., Esteban V., Egido J., Ruiz-Ortega M. (2003). Connective tissue growth factor is a mediator of angiotensin II-induced fibrosis. Circulation.

[28] Rodrigues-Diez R.R., Garcia-Redondo A.B., Orejudo M., Rodrigues-Diez R., Briones A.M., Bosch-Panadero E., Kery G., Pato J., Ortiz A., Salaices M. (2015). The C-terminal module IV of connective tissue growth factor, through EGFR/Nox1 signaling, activates the NF-κB pathway and proinflammatory factors in vascular smooth muscle cells. Antioxid. Redox Signal..

[29] Sánchez-López E., Rayego S., Rodrigues-Díez R., Rodriguez J.S., Rodrigues-Díez R., Rodríguez-Vita J., Carvajal G., Aroeira L.S., Selgas R., Mezzano S.A. (2009). CTGF promotes inflammatory cell infiltration of the renal interstitium by activating NF-κB. J. Am. Soc. Nephrol..

[30] Charrier A., Chen R., Kemper S., Brigstock D.R. (2014). Regulation of pancreatic inflammation by connective tissue growth factor (CTGF/CCN2). Immunology.

[31] Rodrigues-Díez R., Rodrigues-Díez R.R., Rayego-Mateos S., Suarez-Alvarez B., Lavoz C., Stark Aroeira L., Sánchez-López E., Orejudo M., Alique M., Lopez-Larrea C. (2013). The C-terminal module IV of connective tissue growth factor is a novel immune modulator of the Th17 response. Lab. Investig..

[32] Koitabashi N., Arai M., Niwano K., Watanabe A., Endoh M., Suguta M., Yokoyama T., Tada H., Toyama T., Adachi H. (2008). Plasma connective tissue growth factor is a novel potential biomarker of cardiac dysfunction in patients with chronic heart failure. Eur. J. Heart Fail..

[33] Wang X., McLennan S.V., Allen T.J., Twigg S.M. (2010). Regulation of pro-inflammatory and pro-fibrotic factors by CCN2/CTGF in H9c2 cardiomyocytes. J. Cell Commun. Signal..

[34] Hao C., Xie Y., Peng M., Ma L., Zhou Y., Zhang Y., Kang W., Wang J., Bai X., Wang P. (2014). Inhibition of connective tissue growth factor suppresses hepatic stellate cell activation in vitro and prevents liver fibrosis in vivo. Clin. Exp. Med..

[35] Phanish M.K., Winn S.K., Dockrell M.E.C. (2010). Connective tissue growth factor-(CTGF, CCN2)-A marker, mediator and therapeutic target for renal fibrosis. Nephron-Exp. Nephrol..

[36] Ponticos M., Holmes A.M., Shi-wen X., Leoni P., Khan K., Rajkumar V.S., Hoyles R.K., Bou-Gharios G., Black C.M., Denton C.P. (2009). Pivotal role of connective tissue growth factor in lung fibrosis: MAPK-dependent transcriptional activation of type I collagen. Arthritis Rheum..

[37] Shakil Ahmed M., Gravning J., Martinov V.N., von Lueder T.G., Edvardsen T., Czibik G., Moe I.T., Vinge L.E., Øie E., Valen G. (2011). Mechanisms of novel cardioprotective functions of CCN2/CTGF in myocardial ischemia-reperfusion injury. Am. J. Physiol.-Hear. Circ. Physiol..

[38] Gravning J., Ahmed M.S., Von Lueder T.G., Edvardsen T., Attramadal H. (2013). CCN2/CTGF attenuates myocardial hypertrophy and cardiac dysfunction upon chronic pressure-overload. Int. J. Cardiol..

[39] Moe I.T., Ahmed M.S., Stang E., Hagelin E.M.V., Attramadal H. (2016). CTGF/CCN2 postconditioning increases tolerance of murine hearts towards ischemia-reperfusion injury 1ole jørgen kaasbøll. PLoS ONE.

[40] Panek A.N., Posch M.G., Alenina N., Ghadge S.K., Erdmann B., Popova E., Perrot A., Geier C., Morano R.D.I., Bader M. (2009). Connective tissue growth factor overexpression in cardiomyocytes promotes cardiac hypertrophy and protection against pressure overload. PLoS ONE.

[41] Moe I.T., Pham T.A., Hagelin E.M.V., Ahmed M.S., Attramadal H. (2013). CCN2 exerts direct cytoprotective actions in adult cardiac myocytes by activation of the PI3-kinase/Akt/GSK-3β signaling pathway. J. Cell Commun. Signal..

[42] Mori T., Kawara S., Shinozaki M., Hayashi N., Kakinuma T., Igarashi A., Takigawa M., Nakanishi T., Takehara K. (1999). Role and interaction of connective tissue growth factor with transforming growth factor-beta in persistent fibrosis: A mouse fibrosis model. J. Cell. Physiol..

[43] Lasky J., Ortiz L.A., Tonthat B., Hoyle G.W., Corti M., Athas G., Lungarella G., Brody A., Friedman M. (1998). Connective tissue growth factor mRNA expression is upregulated in bleomycin-induced lung fibrosis. Am. J. Physiol..

[44] Strutz F. (2009). Signaling in fibrosis: Targeting the TGF beta, endothelin-1 and CCN2 axis in scleroderma. Front. Biosci. (Elite Ed.).

[45] Bonniaud P., Martin G., Margetts P.J., Ask K., Robertson J., Gauldie J., Kolb M. (2004). Connective tissue growth factor is crucial to inducing a profibrotic environment in “fibrosis-resistant” BALB/c mouse lungs. Am. J. Respir. Cell Mol. Biol..

[46] Bonniaud P., Margetts P.J., Kolb M., Haberberger T., Kelly M., Robertson J., Gauldie J. (2003). Adenoviral gene transfer of connective tissue growth factor in the lung induces transient fibrosis. Am. J. Respir. Crit. Care Med..

[47] Rojas A., Padidam M., Cress D., Grady W.M. (2009). TGF-beta receptor levels regulate the specificity of signaling pathway activation and biological effects of TGF-beta. Biochim. Biophys. Acta.

[48] Lönn P., Morén A., Raja E., Dahl M., Moustakas A. (2009). Regulating the stability of TGFbeta receptors and Smads. Cell Res..

[49] Kang J.S., Liu C., Derynck R. (2009). New regulatory mechanisms of TGF-beta receptor function. Trends Cell Biol..

[50] Rayego-Mateos S., Rodrigues-Díez R., Morgado-Pascual J.L., Rodrigues Díez R.R., Mas S., Lavoz C., Alique M., Pato J., Keri G., Ortiz A. (2013). Connective tissue growth factor is a new ligand of epidermal growth factor receptor. J. Mol. Cell Biol..

[51] Zhang Y., Alexander P.B., Wang X.F. (2017). TGF-β Family Signaling in the Control of Cell Proliferation and Survival. Cold Spring Harb. Perspect. Biol..

[52] Rayego-Mateos S., Campillo S., Rodrigues-Diez R.R., Tejera-Muñoz A., Marquez-Exposito L., Goldschmeding R., Rodríguez-Puyol D., Calleros L., Ruiz-Ortega M. (2021). Interplay between extracellular matrix components and cellular and molecular mechanisms in kidney fibrosis. Clin. Sci. (Lond.).

[53] Chen Z., Zhang N., Chu H.Y., Yu Y., Zhang Z.K., Zhang G., Zhang B.T. (2020). Connective Tissue Growth Factor: From Molecular Understandings to Drug Discovery. Front. Cell Dev. Biol..

[54] Di Guglielmo G.M., Le Roy C., Goodfellow A.F., Wrana J.L. (2003). Distinct endocytic pathways regulate TGF-beta receptor signalling and turnover. Nat. Cell Biol..

[55] McCaffrey T.A., Consigli S., Du B., Falcone D.J., Sanborn T.A., Spokojny A.M., Bush H.L. (1995). Decreased type II/type I TGF-beta receptor ratio in cells derived from human atherosclerotic lesions. Conversion from an antiproliferative to profibrotic response to TGF-beta1. J. Clin. Investig..

[56] McCaffrey T.A., Du B., Fu C., Bray P.J., Sanborn T.A., Deutsch E., Tarazona N., Shaknovitch A., Newman G., Patterson C. (1999). The expression of TGF-beta receptors in human atherosclerosis: Evidence for acquired resistance to apoptosis due to receptor imbalance. J. Mol. Cell. Cardiol..

[57] McCaffrey T.A., Du B., Consigli S., Szabo P., Bray P.J., Hartner L., Weksler B.B., Sanborn T.A., Bergman G., Bush H.L. (1997). Genomic instability in the type II TGF-beta1 receptor gene in atherosclerotic and restenotic vascular cells. J. Clin. Investig..

[58] Rodríguez-Vita J., Sínchez-Galín E., Santamaría B., Sánchez-López E., Rodrigues-Díez R., Blanco-Colio L.M., Egido J., Ortiz A., Ruiz-Ortega M. (2008). Essential role of TGF-beta/Smad pathway on statin dependent vascular smooth muscle cell regulation. PLoS ONE.

[59] Chaqour B. (2020). Caught between a “Rho” and a hard place: Are CCN1/CYR61 and CCN2/CTGF the arbiters of microvascular stiffness?. J. Cell Commun. Signal..

[60] Kubota S., Takigawa M. (2015). Cellular and molecular actions of CCN2/CTGF and its role under physiological and pathological conditions. Clin. Sci. (Lond.).

[61] Wang R., Xu Y.J., Liu X.S., Zeng D.X., Xiang M. (2011). Knockdown of connective tissue growth factor by plasmid-based short hairpin RNA prevented pulmonary vascular remodeling in cigarette smoke-exposed rats. Arch. Biochem. Biophys..

[62] Szabó Z., Magga J., Alakoski T., Ulvila J., Piuhola J., Vainio L., Kivirikko K.I., Vuolteenaho O., Ruskoaho H., Lipson K.E. (2014). Connective tissue growth factor inhibition attenuates left ventricular remodeling and dysfunction in pressure overload-induced heart failure. Hypertension.

[63] Gravning J., Ørn S., Kaasbøll O.J., Martinov V.N., Manhenke C., Dickstein K., Edvardsen T., Attramadal H., Ahmed M.S. (2012). Myocardial connective tissue growth factor (CCN2/CTGF) attenuates left ventricular remodeling after myocardial infarction. PLoS ONE.

[64] Ivkovic S., Yoon B.S., Popoff S.N., Safadi F.F., Libuda D.E., Stephenson R.C., Daluiski A., Lyons K.M. (2003). Connective tissue growth factor coordinates chondrogenesis and angiogenesis during skeletal development. Development.

[65] Rayego-Mateos S., Morgado-Pascual J.L., Rodrigues-Diez R.R., Rodrigues-Diez R., Falke L.L., Mezzano S., Ortiz A., Egido J., Goldschmeding R., Ruiz-Ortega M. (2018). Connective tissue growth factor induces renal fibrosis via epidermal growth factor receptor activation. J. Pathol..

[66] Fontes M.S.C., Kessler E.L., van Stuijvenberg L., Brans M.A., Falke L.L., Kok B., Leask A., van Rijen H.V.M., Vos M.A., Goldschmeding R. (2015). CTGF knockout does not affect cardiac hypertrophy and fibrosis formation upon chronic pressure overload. J. Mol. Cell. Cardiol..

[67] Rodrigues-Díez R.R., Tejera-Muñoz A., Esteban V. (2022). CCN2 (Cellular Communication Network Factor 2) Deletion Alters Vascular Integrity and Function Predisposing to Aneurysm Formation. Hypertension.

[68] Chen X., Rateri D.L., Howatt D.A., Balakrishnan A., Moorleghen J.J., Cassis L.A., Daugherty A. (2016). TGF-β neutralization enhances angii-induced aortic rupture and aneurysm in both thoracic and abdominal regions. PLoS ONE.

[69] Mallat Z., Ait-Oufella H., Tedgui A. (2017). The Pathogenic Transforming Growth Factor-β Overdrive Hypothesis in Aortic Aneurysms and Dissections: A Mirage?. Circ. Res..

[70] Lareyre F., Clment M., Raffort J., Pohlod S., Patel M., Esposito B., Master L., Finigan A., Vandestienne M., Stergiopulos N. (2017). TGFβ (transforming growth factor-β) blockade induces a human-like disease in a nondissecting mouse model of abdominal aortic aneurysm. Arterioscler. Thromb. Vasc. Biol..

[71] Rodriguez-Vita J., Sanchez-Lopez E., Esteban V., Ruperez M., Egido J., Ruiz-Ortega M. (2005). Angiotensin II activates the Smad pathway in vascular smooth muscle cells by a transforming growth factor-beta-independent mechanism. Circulation.

[72] Jinnin M., Ihn H., Tamaki K. (2006). Characterization of SIS3, a novel specific inhibitor of Smad3, and its effect on transforming growth factor-beta1-induced extracellular matrix expression. Mol. Pharmacol..

[73] Herbertz S., Sawyer J.S., Stauber A.J., Gueorguieva I., Driscoll K.E., Estrem S.T., Cleverly A.L., Desaiah D., Guba S.C., Benhadji K.A. (2015). Clinical development of galunisertib (LY2157299 monohydrate), a small molecule inhibitor of transforming growth factor-beta signaling pathway. Drug Des. Devel. Ther..

